# Supplementary material for: Synthesis and DNase I Inhibitory Properties of New Squaramides
Source: Molecules. 2023 Jan 5;28(2):538. doi: 10.3390/molecules28020538 (PMC9863136; doi:10.3390/molecules28020538)
Supplement: Supplementary file 1 [file molecules-28-00538-s001.zip › Tables.pdf]

Table S1. Bond Lengths for compound **3a** and **3c**

Compound **3a**			Compound **3c**		
**Bond**		**Length/Å**	**bond**		**Length/Å**
O2	C2	1.322(2)	O2	C2	1.317(3)
O2	C12	1.456(2)	O2	C12	1.451(3)
O3	C3	1.222(2)	N1	C5	1.467(3)
C7	C8	1.379(2)	N1	C1	1.318(3)
C7	C6	1.501(2)	O4	C4	1.208(3)
C7	C11	1.377(3)	N2	C8	1.335(3)
N1	C1	1.319(2)	N2	C9	1.332(3)
N1	C5	1.452(2)	O3	C3	1.224(3)
C1	C2	1.397(2)	C7	C8	1.380(3)
C1	C4	1.469(3)	C7	C11	1.382(3)
C2	C3	1.442(3)	C7	C6	1.503(3)
N2	C8	1.332(2)	C2	C1	1.394(3)
N2	C9	1.329(3)	C2	C3	1.459(3)
C3	C4	1.507(3)	C5	C6	1.524(3)
O4	C4	1.209(2)	C5	C14	1.517(3)
C5	C6	1.520(3)	C1	C4	1.476(3)
C11	C10	1.375(3)	C11	C10	1.379(4)
C9	C10	1.366(3)	C4	C3	1.500(4)
C12	C13	1.452(3)	C10	C9	1.360(4)
			C12	C13	1.446(4)

Table S2. Bond Angles for compound **3a** and **3c**

Compound **3a**				Compound **3c**			
**Atom**	**Atom**	**Atom**	**Angle/°**	**Atom**	**Atom**	**Atom**	**Angle/°**
C2	O2	C12	116.13(14)	**Atom**	**Atom**	**Atom**	**Angle/°**
C8	C7	C6	121.04(16)	C2	O2	C12	116.64(19)
C11	C7	C8	116.59(17)	C1	N1	C5	123.97(19)
C11	C7	C6	122.34(16)	C9	N2	C8	116.6(2)
C1	N1	C5	124.41(16)	C8	C7	C11	116.8(2)
N1	C1	C2	137.29(18)	C8	C7	C6	120.6(2)
N1	C1	C4	132.46(17)	C11	C7	C6	122.6(2)
C2	C1	C4	90.24(15)	O2	C2	C1	130.0(2)
O2	C2	C1	129.66(16)	O2	C2	C3	136.4(2)
O2	C2	C3	136.19(16)	C1	C2	C3	93.54(19)
C1	C2	C3	94.11(15)	N2	C8	C7	124.7(2)
C9	N2	C8	116.94(17)	N1	C5	C6	110.85(19)
O3	C3	C2	137.57(19)	N1	C5	C14	109.55(18)
O3	C3	C4	135.36(19)	C14	C5	C6	110.80(19)
C2	C3	C4	87.06(15)	N1	C1	C2	137.4(2)
N2	C8	C7	124.48(18)	N1	C1	C4	132.0(2)
N1	C5	C6	112.47(16)	C2	C1	C4	90.61(18)
C7	C6	C5	112.27(15)	C10	C11	C7	119.2(2)

C10	C11	C7	120.14(18)	O4	C4	C1	134.3(2)
N2	C9	C10	123.43(19)	O4	C4	C3	137.0(2)
C9	C10	C11	118.38(19)	C1	C4	C3	88.63(18)
C1	C4	C3	88.57(15)	O3	C3	C2	136.8(3)
O4	C4	C1	134.92(19)	O3	C3	C4	136.0(2)
O4	C4	C3	136.51(19)	C2	C3	C4	87.21(18)
C13	C12	O2	108.63(17)	C7	C6	C5	114.37(18)
				C9	C10	C11	119.3(2)
				N2	C9	C10	123.4(2)
				C13	C12	O2	108.9(2)

Table S3. Hydrogen Bonding and weak interactions for **3a** and **3c**.

				**3a**		
**D**	**H**	**A**	**d(D-H)/Å**	**d(H-A)/Å**	**d(D-A)/Å**	**D-H-A/°**
N1	H1	N2[1]	0.86	2.05	2.908(2)	172.4
C5	H5A	O2	0.97	2.62	3.283(2)	126.1
C5	H5B	O4[2]	0.97	2.46	3.408(2)	166.5
C6	H6B	O3[3]	0.97	2.46	3.385(2)	159.1
C13	H13B	O3[4]	0.96	2.64	3.587(3)	167.4

[1]1+X,+Y,+Z; [2]3/2-X,-1/2+Y,1/2-Z; [3]1/2-X,-1/2+Y,1/2-Z; [4]-X,1-Y,1-Z

				**3c**		
**D**	**H**	**A**	**d(D-H)/Å**	**d(H-A)/Å**	**d(D-A)/Å**	**D-H-A/°**
C5	H5	O2	0.98	2.59	3.279(3)	127.5
C14	H14B	O3[1]	0.96	2.64	3.587(3)	169.8
C13	H13C	O3[2]	0.96	2.41	3.365(4)	170.6
N1	H1	N2[3]	0.95(3)	1.98(3)	2.927(3)	177(3)

[1]+X,-1+Y,+Z; [2]2-X,1-Y,2-Z; [3]-1+X,+Y,+Z
