# Supplementary figures and images for: Synthesis and DNase I Inhibitory Properties of New Squaramides
Source: Molecules. 2023 Jan 5;28(2):538. doi: 10.3390/molecules28020538 (PMC9863136; doi:10.3390/molecules28020538)

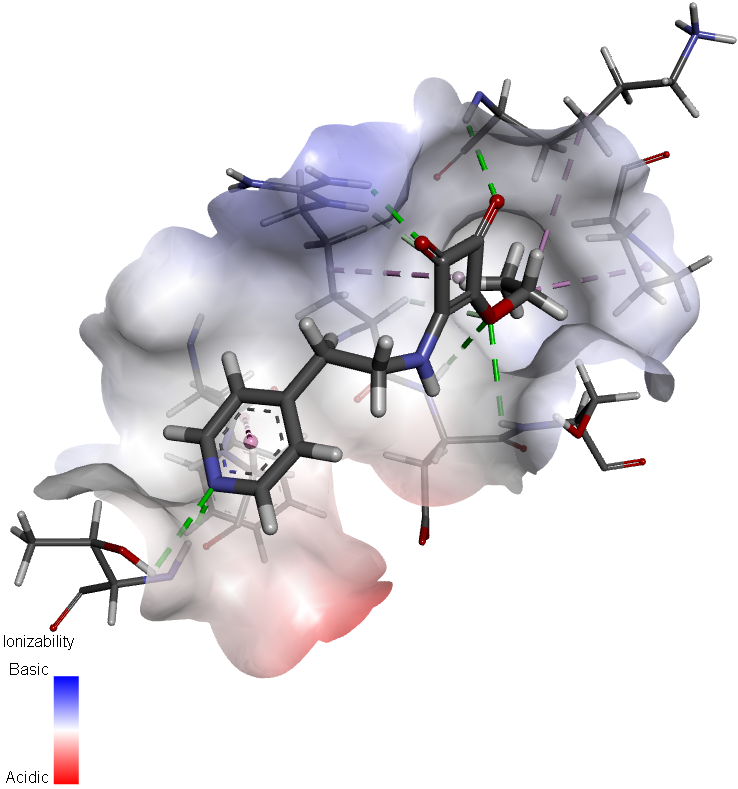

Supplement: Supplementary file 1 [file molecules-28-00538-s001.zip › Figure_S1_A.tiff]

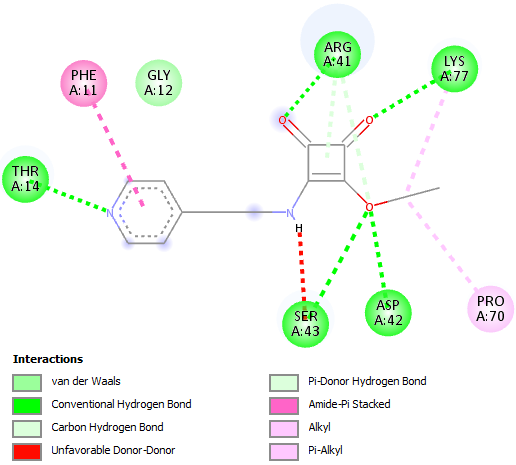

Supplement: Supplementary file 1 [file molecules-28-00538-s001.zip › Figure_S1_B.tiff]

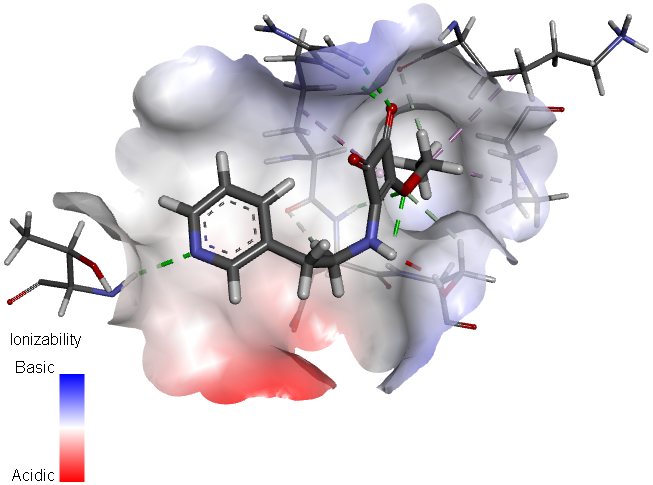

Supplement: Supplementary file 1 [file molecules-28-00538-s001.zip › Figure_S1_C.tiff]

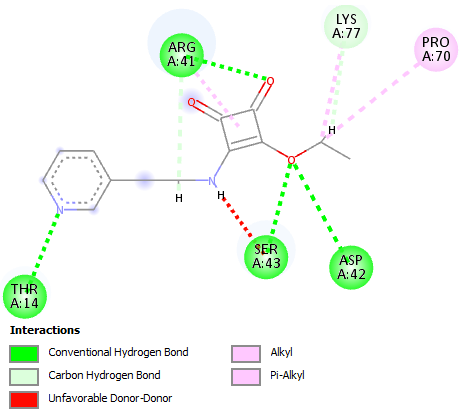

Supplement: Supplementary file 1 [file molecules-28-00538-s001.zip › Figure_S1_D.tiff]

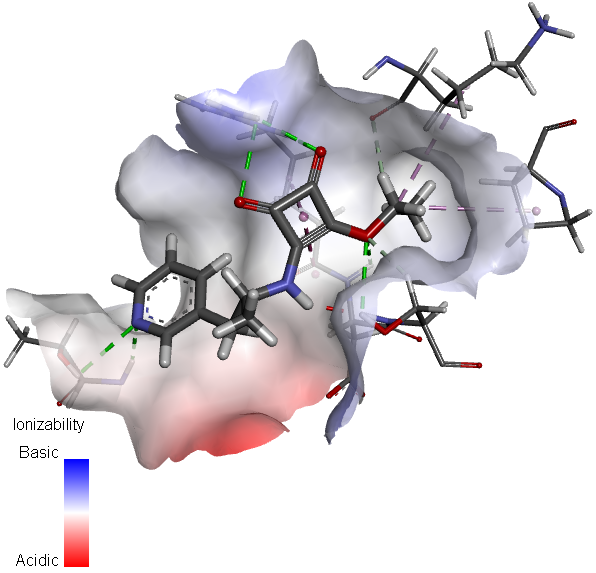

Supplement: Supplementary file 1 [file molecules-28-00538-s001.zip › Figure_S1_E.tiff]

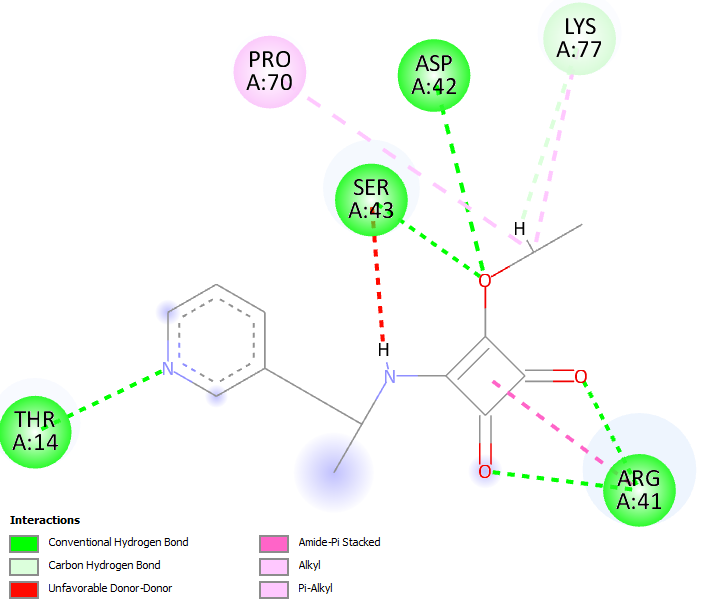

Supplement: Supplementary file 1 [file molecules-28-00538-s001.zip › Figure_S1_F.tiff]

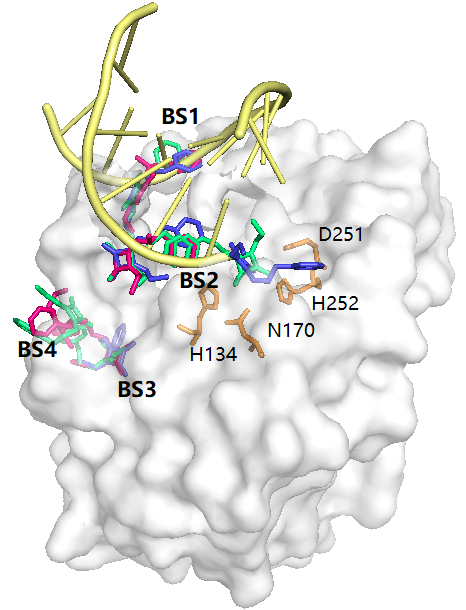

Supplement: Supplementary file 1 [file molecules-28-00538-s001.zip › Figure_S2.tiff]

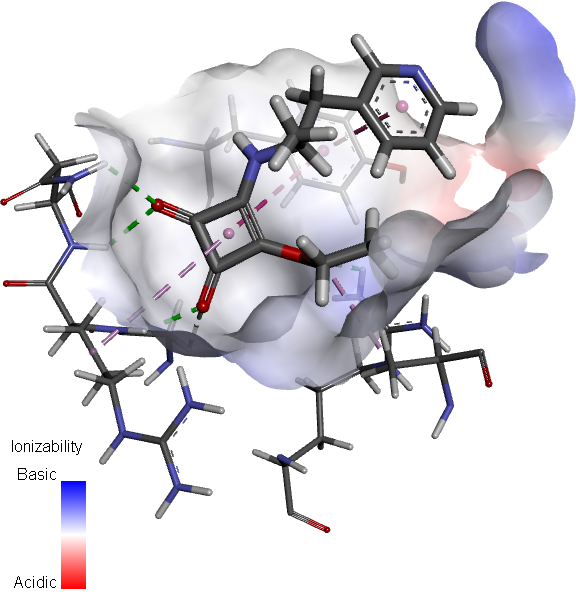

Supplement: Supplementary file 1 [file molecules-28-00538-s001.zip › Figure_S3_A.tiff]

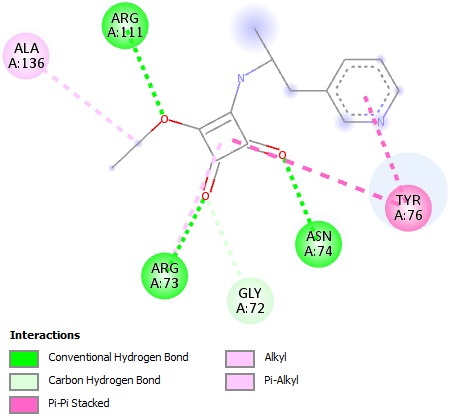

Supplement: Supplementary file 1 [file molecules-28-00538-s001.zip › Figure_S3_B.tiff]

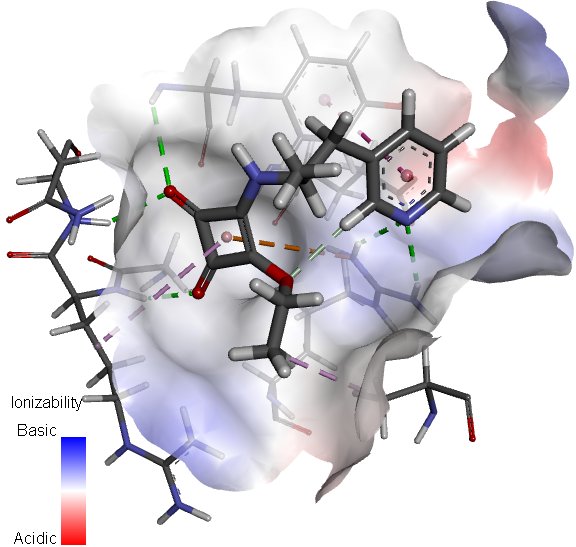

Supplement: Supplementary file 1 [file molecules-28-00538-s001.zip › Figure_S3_C.tiff]

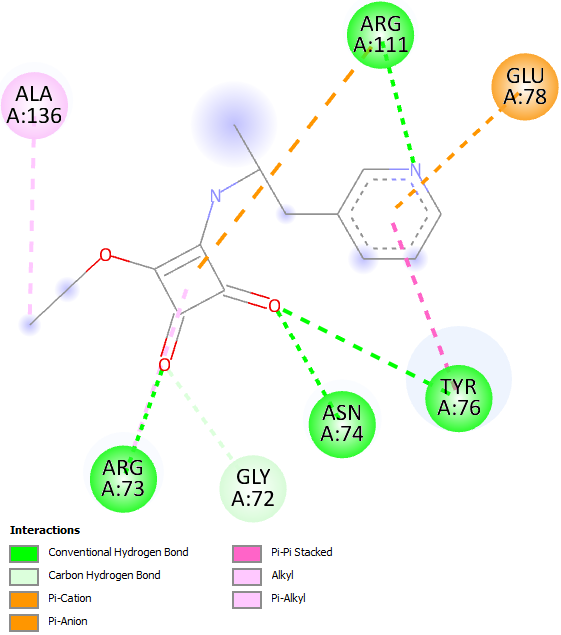

Supplement: Supplementary file 1 [file molecules-28-00538-s001.zip › Figure_S3_D.tiff]

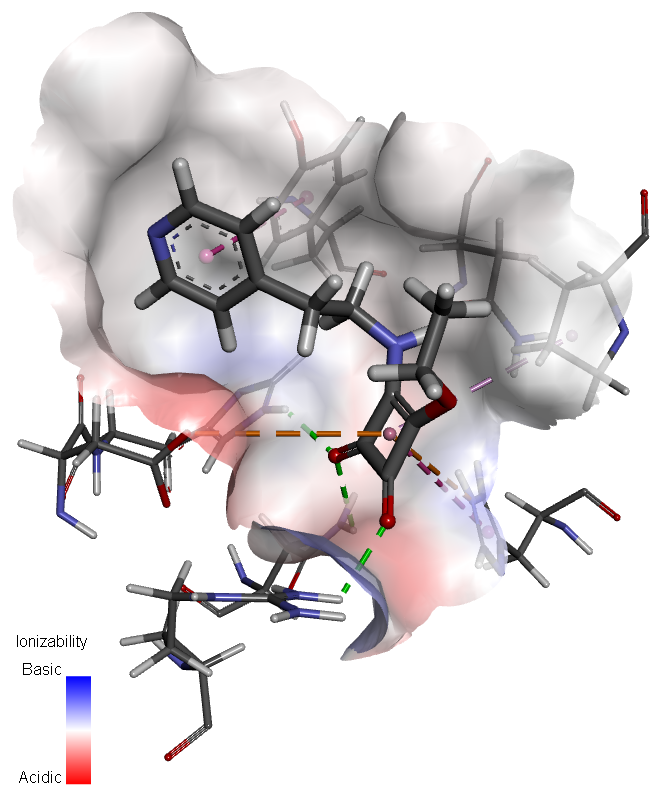

Supplement: Supplementary file 1 [file molecules-28-00538-s001.zip › Figure_S4_A.tiff]

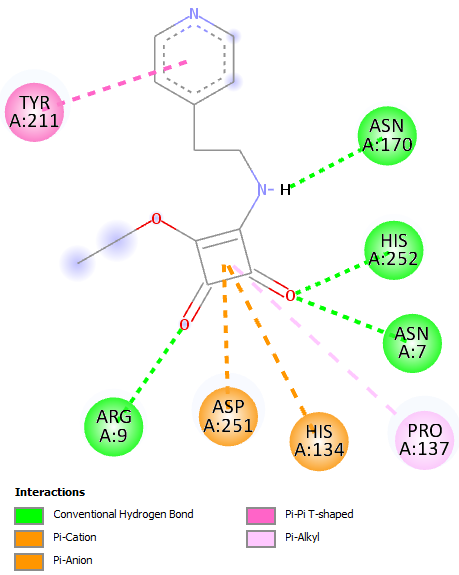

Supplement: Supplementary file 1 [file molecules-28-00538-s001.zip › Figure_S4_B.tiff]

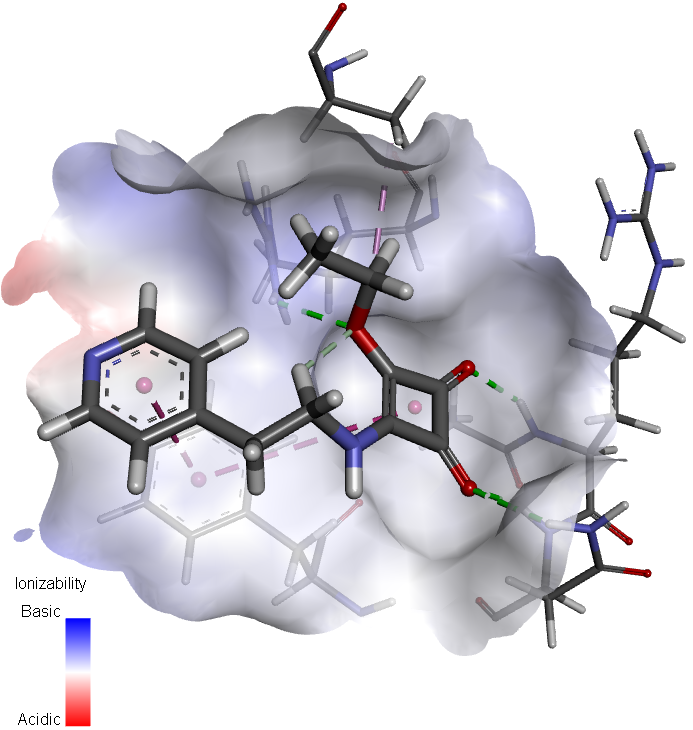

Supplement: Supplementary file 1 [file molecules-28-00538-s001.zip › Figure_S4_C.tiff]

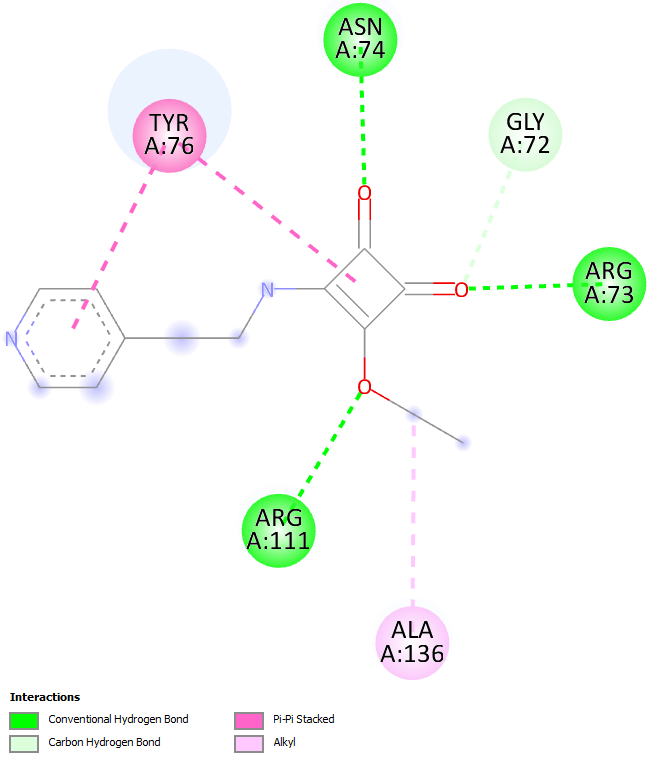

Supplement: Supplementary file 1 [file molecules-28-00538-s001.zip › Figure_S4_D.tiff]

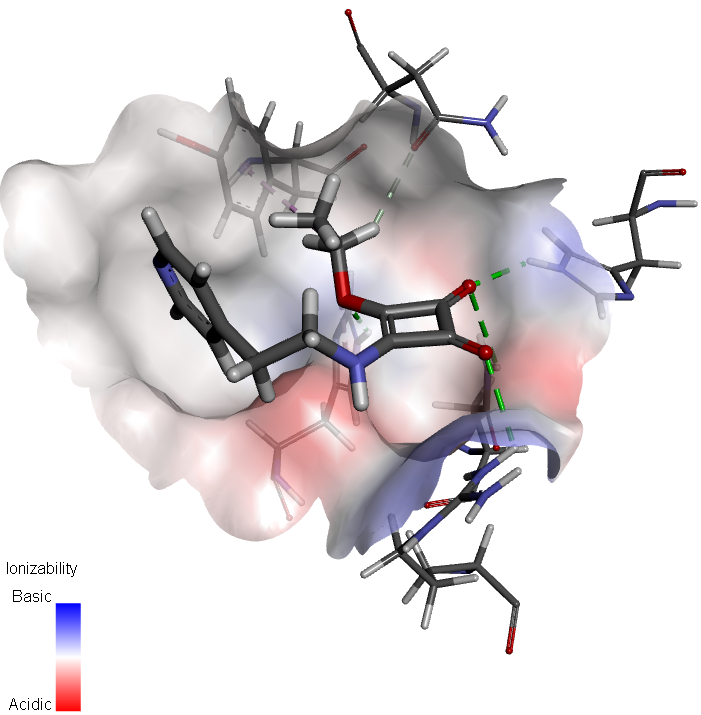

Supplement: Supplementary file 1 [file molecules-28-00538-s001.zip › Figure_S4_E.tiff]

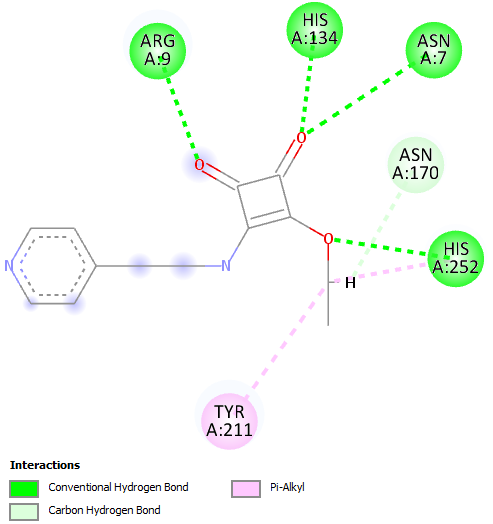

Supplement: Supplementary file 1 [file molecules-28-00538-s001.zip › Figure_S4_F.tiff]

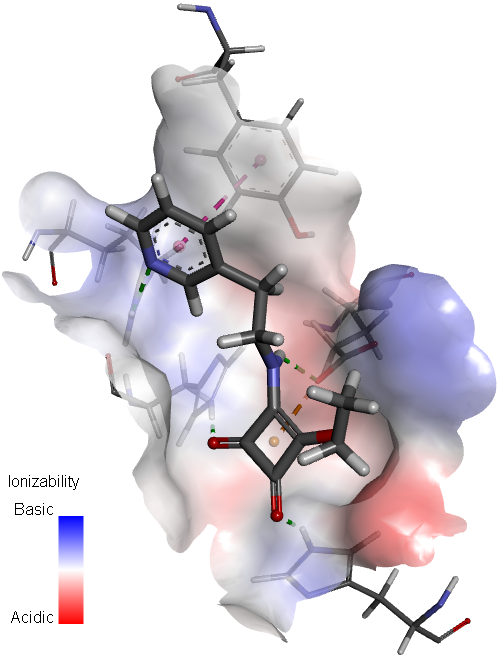

Supplement: Supplementary file 1 [file molecules-28-00538-s001.zip › Figure_S5_A.tiff]

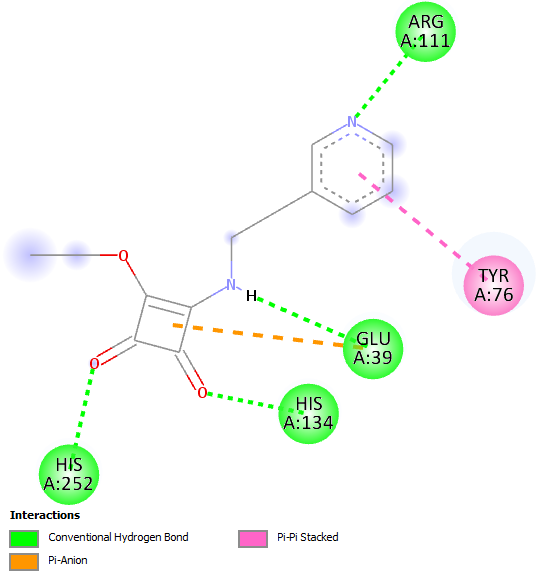

Supplement: Supplementary file 1 [file molecules-28-00538-s001.zip › Figure_S5_B.tiff]

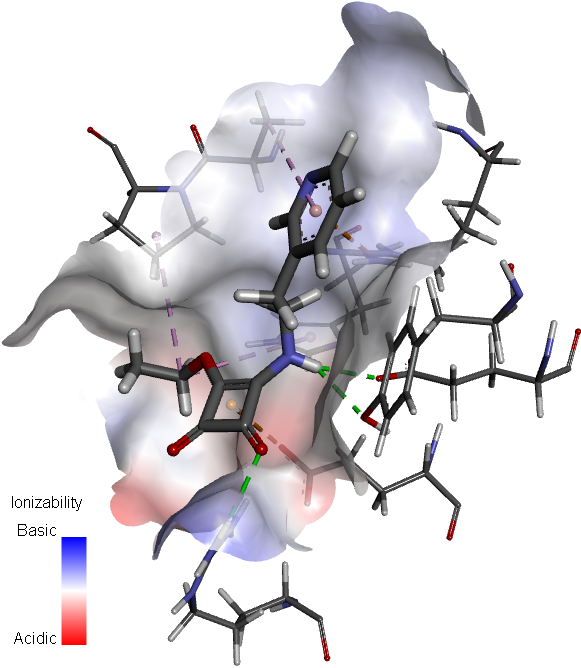

Supplement: Supplementary file 1 [file molecules-28-00538-s001.zip › Figure_S5_C.tiff]

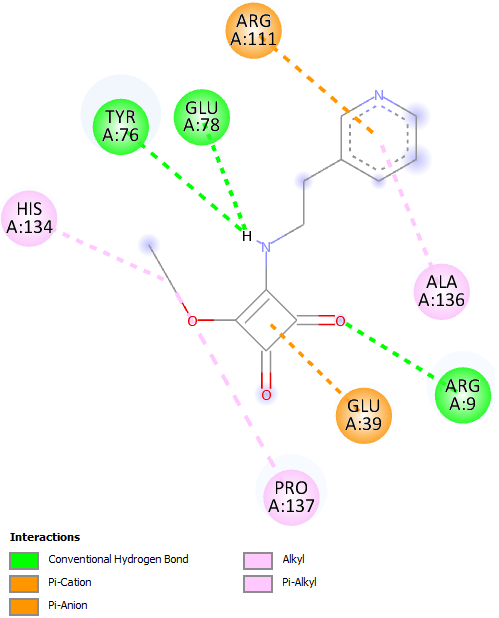

Supplement: Supplementary file 1 [file molecules-28-00538-s001.zip › Figure_S5_D.tiff]

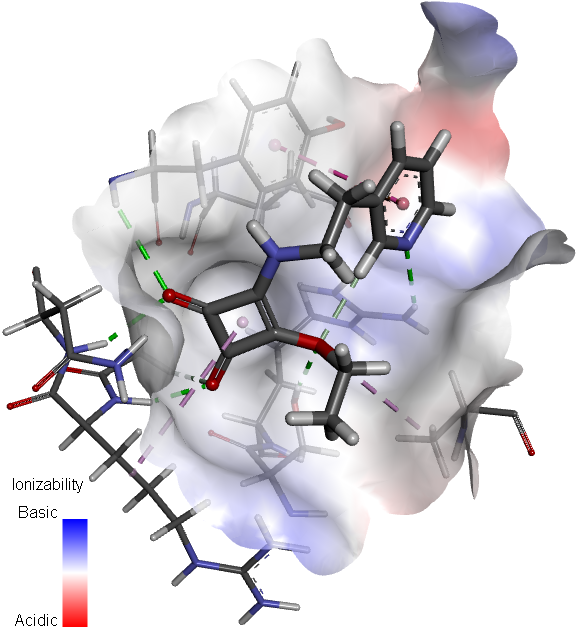

Supplement: Supplementary file 1 [file molecules-28-00538-s001.zip › Figure_S5_E.tiff]

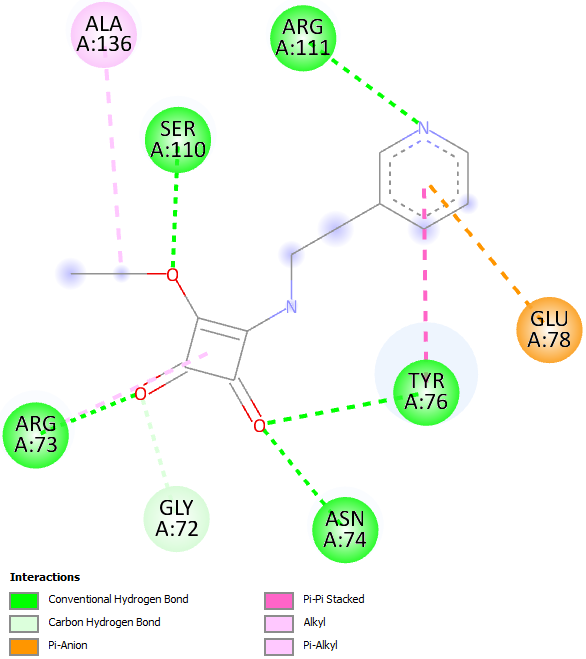

Supplement: Supplementary file 1 [file molecules-28-00538-s001.zip › Figure_S5_F.tiff]

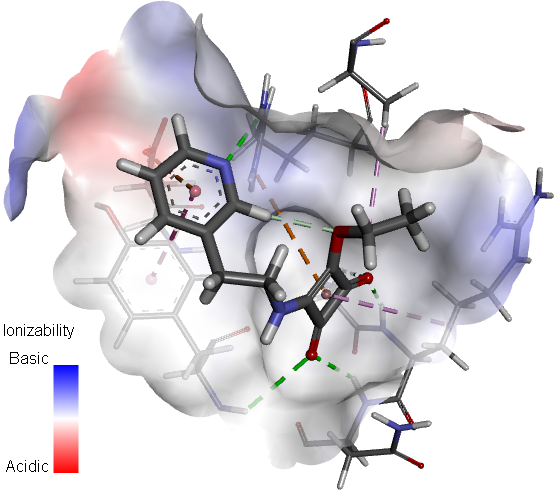

Supplement: Supplementary file 1 [file molecules-28-00538-s001.zip › Figure_S5_G.tiff]

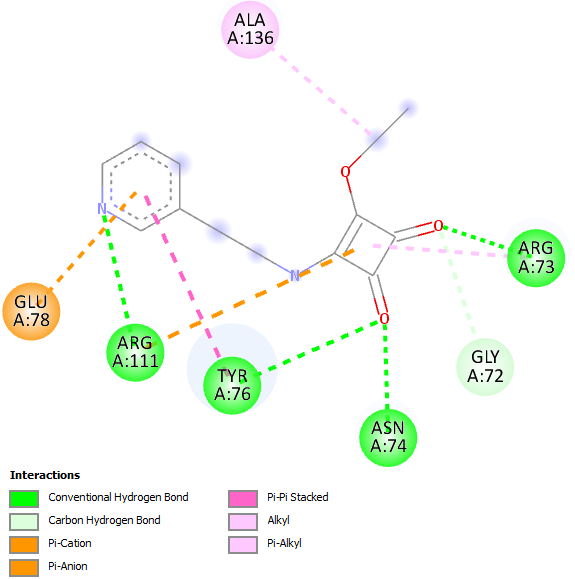

Supplement: Supplementary file 1 [file molecules-28-00538-s001.zip › Figure_S5_H.tiff]
